# A Meta-Analysis of Efficacy and Safety of Infliximab for Prevention of Postoperative Recurrence in Patients with Crohn's Disease

**DOI:** 10.1155/2018/2615978

**Published:** 2018-12-13

**Authors:** He Huang, Su Xu, Fubin Huang, Xia Wang, Yong Chen, Zhaoshan Xu

**Affiliations:** ^1^Department of Gastroenterology, Yancheng Hospital of Traditional Chinese Medicine affiliated to Nanjing University of Chinese Medicine, Yancheng 224001, China; ^2^Department of Anorectal Surgery, Yancheng Hospital of Traditional Chinese Medicine affiliated to Nanjing University of Chinese Medicine, Yancheng 224001, China

## Abstract

**Objective:**

We sought to investigate the efficacy and safety of Infliximab for prevention of postoperative recurrence in patients with Crohn's disease (CD), in a meta-analysis of clinical trial results.

**Methods:**

The Medline, Embase, PubMed, and Web of Science databases were systematically searched for suitable studies. A meta-analysis of enrolled studies was performed to analyze the efficacy of Infliximab on outcomes regarding the prevention of postoperative recurrence of CD. A Galbraith radial plot was used to quantify the heterogeneity. Funnel plot and Egger test were performed to describe the bias of publication. A Forest plot was prepared to indicate the efficacy outcomes.

**Results:**

A total of 7 prospective trials were included in our meta-analysis (N=455). The Funnel plot and Egger test showed there was no significant bias in the included publications. The Cochrane collaboration tool indicated that all 7 prospective trials were of high quality. The results of Galbraith radial plot showed that no study was the source of heterogeneity. Compared with the placebo group, Infliximab decreased the rates of endoscopic recurrence (RR =0.421; 95% CI 0.328 to 0.539;* p*<0.001), and there was a significant reduction in rates of clinical recurrence in the Infliximab-treated group (RR =0.519; 95% CI 0.349 to 0.774;* p*=0.001). Furthermore, Infliximab treatment did not show adverse effects as other systematic therapeutic drugs, indicating that Infliximab treatment is effective and well tolerated.

**Conclusion:**

Compared with the controls, Infliximab is a promising therapeutic agent for the management of CD patients.

## 1. Introduction

Crohn's disease (CD), a chronic inflammatory disorder occurred in gastrointestinal tract that may cause serious tissue damage, is one of inflammatory bowel diseases [1]. CD usually significantly affects the patients' quality of life. In some severe cases, CD poses challenges for the entire family and may lead to psychological problems, even a substantial economic burden, as it frequently occurs in young patients [2]. At present, over 75% of CD patients need surgical resection for penetrating and structuring complications, and ileocolonic resection is the most common therapeutic strategy for such condition [3].

However, surgery is not once for all and CD patients often have clinical or endoscopic recurrence within 2-6 years of resection in up to 41% and 89%, respectively [4]. Some studies have proved the efficacy of traditional drugs in the prevention of postoperative recurrence of CD, with different results [5–8].

Owing to the efficacy of biological treatment in the induction and maintenance of CD, multiple studies have been conducted to find out the efficacy of antitumor necrosis factor (anti-TNF) agents in the prevention of postoperative recurrence in CD [9–13]. The anti-TNF agents were in fact shown to be more effective than conventional treatment in the prevention of postoperative clinical and endoscopic recurrence [14–18]. Furthermore, three recent meta-analyses have put forward the conclusion above [19–21]. However, there are few studies researching the efficacy of Infliximab, one of the anti-TNF agents, for the prevention of postoperative recurrence in CD, and there is currently no conclusive evidence of the superiority of Infliximab therapy in this setting. Hence, a meta-analysis of prospective trials was performed to compare the efficacy and safety of Infliximab in the prevention of postoperative recurrence of CD after ileocolonic resection.

In the present study, we sought to analyze the efficacy and safety of Infliximab for prevention of recurrence in patients with CD in a meta-analysis of clinical trial results. Suitable trials were selected by searching the PubMed, Medline, Embase, and Web of Science databases; only prospective trials were included. The results revealed that Infliximab treatment yielded a significant improvement in the rates of CD recurrence and did not increase the adverse event occurrence, indicating that Infliximab treatment is a promising therapeutic strategy for the management of CD patients.

## 2. Methods

This meta-analysis was performed in accordance with the guidelines of the Preferred Reporting Items for Systematic Reviews and Meta-Analyses (PRISMA) statement (http://www.prisma-statement.org/).

### 2.1. Search Strategy and Inclusion Criteria

On Jun 1^st^, 2018, we conducted a systematic search of PubMed, Medline, Embase, and Web of Science databases for randomized controlled trials or cohort studies published between Jun 1^st^, 2000 and Jun 1^st^, 2018 in English using the search terms “Infliximab” and “Crohn's disease”. All the studies enrolled using this strategy were checked independently by two authors; the articles that met all inclusion criteria were enrolled in the meta-analysis. The inclusion criteria were as follows: (1) patients diagnosed with CD based on tissue biopsy and treated with Infliximab; (2) double-blind, randomized, placebo-controlled or prospective studies; and (3) the outcome measures regarding the rates of CD recurrence.

### 2.2. Testing for Heterogeneity and Risk of Bias, and Sensitivity Analysis

A Q and I^2^ test were performed to analyze the heterogeneity of the studies included in this meta-analysis. A Galbraith radial plot was produced to indicate the cause of heterogeneity in studies. Each study was represented as a single dot, with a central regression line through the plot. The Cochrane collaboration tool method was used to evaluate the quality of the included studies and the risk of bias, and the Egger test was performed, a linear regression method to evaluate the bias in publications in the meta-analysis. Sensitivity analysis was calculated to evaluate the stability and reliability of the results.

### 2.3. Data Extraction and Outcome Measures

In our systematic review of the literature, the following variables were extracted. Name of the study, drug regimen, type of ileocolonic resection, timing of initiation of treatment, recurrence definition and follow-up were included. The aim of the meta-analysis was to investigate the efficacy and safety of Infliximab for the prevention of recurrence in patients with CD across studies. The outcomes included in the meta-analysis were the rates of endoscopic recurrence and clinical recurrence.

### 2.4. Statistical Analysis

From the included studies in the analysis, we extracted the rates of endoscopic recurrence and clinical recurrence for Infliximab and the placebo, along with the corresponding 95% CI. The overall summary effect sizes were estimated using a random-effects model if heterogeneity was >50%, and fixed-effects model was used if heterogeneity was <50%. Numeration data was presented as relative risk (RR) and a p value of <0.05 was considered to be statistically significant. Forest plots and data analysis were performed using Review Manager (RevMan version 5.3; Nordic Cochrane Centre, Copenhagen, Denmark) and STATA software (version 12.0; StataCorp, College Station, TX, USA).

## 3. Results

### 3.1. Study Selection

**Figure 1:**
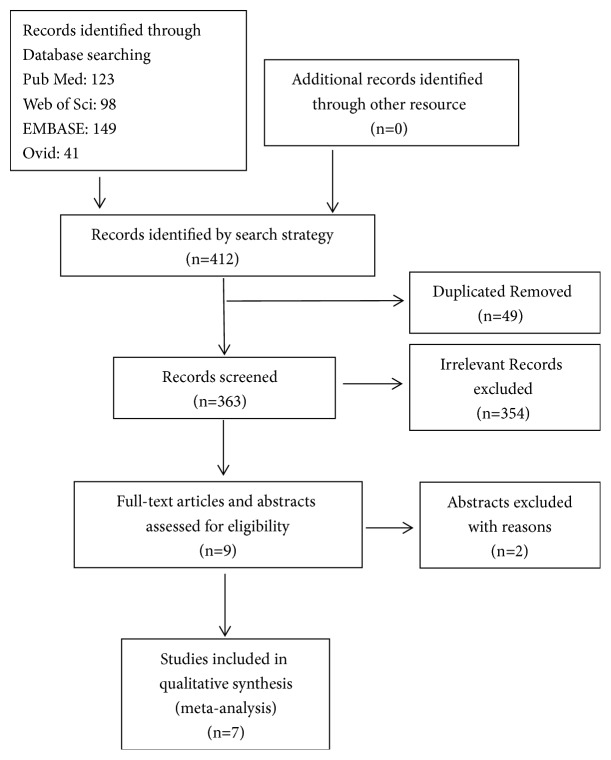
Flow diagram of study selection. A total of 455 potentially relevant studies were collected, of which 7 prospective trials were included in the meta-analysis.

A search of the PubMed, MEDLINE, Embase, and Web of Science databases was conducted, and 7 prospective trials were enrolled, after excluding duplicated, irrelevant, and nonfull text articles. The systematic search identified 413 original articles; the flow diagram for the identified studies is shown in Figure 1. A total of 49 duplicates, 2 abstracts, and 354 irrelevant articles were excluded as they did not meet the inclusion criteria for this meta-analysis. Finally, 7 prospective trials were included in the meta-analysis. All studies reported quantitative analysis regarding the Infliximab in patients with CD. All were published in English.

### 3.2. Study Characterization

This systematic review included the data of 455 patients diagnosed with CD. A total of 7 prospective trials were enrolled in the systematic review. All studies had control groups in which the baseline of CD severity did not significantly differ from the treatment group. The characteristics of the cohort studies are shown in Table 1. In the 7 prospective trials, all the studies considered Infliximab monotherapy, and all the patients in the treatment group received Infliximab 5mg/kg once a day. The timing of initiation of treatment ranged from 2 to 8 weeks. Sorrentino et al. (2007) and Fukushima et al. (2018) were designed for 24 months, and other trials were designed for 12 months. In Sorrentino et al. (2007), Regueiro et al. (2009), Yoshida et al. (2012), and Regueiro et al. (2016) trials, the type of ileocolonic resection was macroscopically diseased bowel resected, and in Armuzzi et al. (2013), Tursi et al. (2014), and Fukushima et al. (2018) studies, curative ileocolonic resection was made. Moreover, the clinical recurrence definition in the 7 studies was based on Hanauer, CDAI or HBI criteria, while the endoscopic recurrence of all the trials was defined as Rutgeerts score≥2. The characteristics of the included trials are shown in Table 1.

### 3.3. Risk of Bias, Sensitivity Analysis, and Quality Assessment

**Figure 2:**
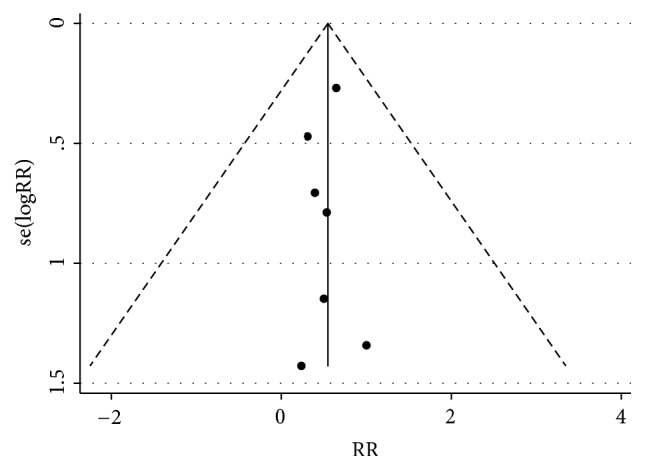
Risk of publication bias for each trial. Both the Funnel plot and Egger test showed no significant evidence of bias in the publication.

**Figure 3:**
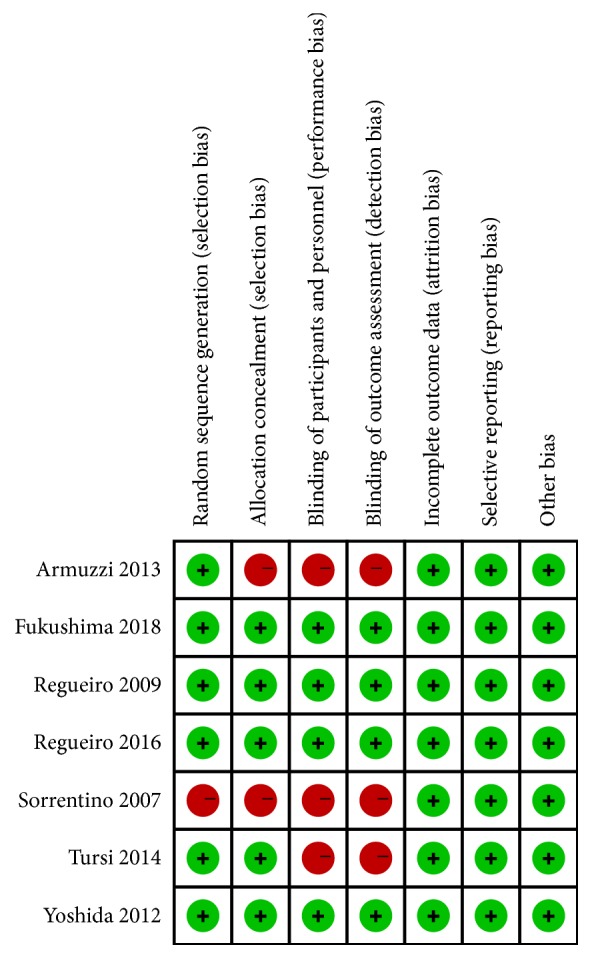
Risk of bias assessment. The qualities of the included studies were evaluated using Cochrane collaboration tool, indicating that there is no significant risk of bias in the included trials of this meta-analysis.

**Figure 4:**
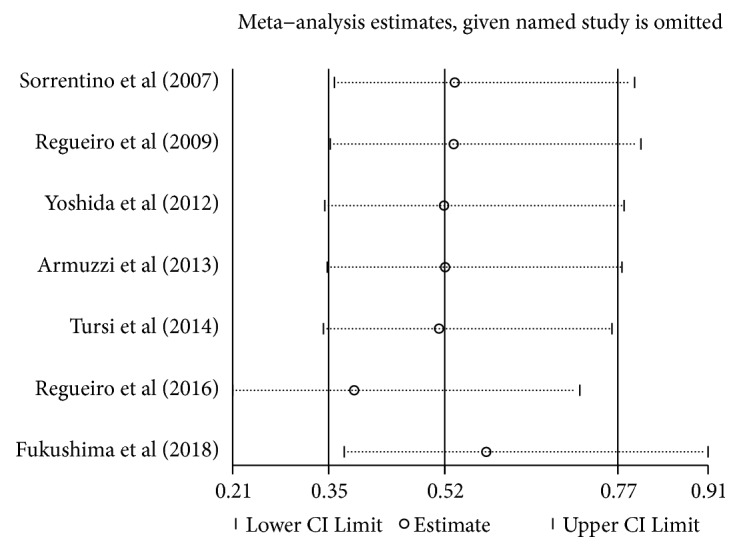
Sensitivity analysis plot. Hollow circles stand for relative risk (RR) of included trials, and Horizontal lines represent 95%Cls of the RR. Almost all the trials were within the two regression lines.

A Funnel plot was produced to describe the bias in publication of the 7 prospective trials (Figure 2). All studies enrolled in the meta-analysis exhibited a low risk of bias. The Cochrane collaboration tool was used to identify the risk of bias of 7 prospective trials, as shown in Figure 3. All studies applied random sequence generation, allocation concealment, blinding of participants and personnel, and blinding of outcome assessment and had a low risk of incomplete outcome data and selective reporting. The clinical outcome data in the included studies were based on existing guidelines, and their baselines were similar. A Funnel plot and Egger test showed there was no significant bias in the cohort studies (p>0.05). A sensitivity analysis plot was performed to analyze the sensitivity of the trials included in this meta-analysis, which indicated that no studies needed to be excluded (Figure 4).

### 3.4. Efficacy Outcomes of Infliximab

In a pooled analysis of the 7 prospective trials, a significant improvement in efficacy for Infliximab versus the placebo was observed in various clinical indexes: rates of endoscopic recurrence and clinical recurrence.

#### 3.4.1. Rates of Endoscopic Recurrence

**Figure 5:**
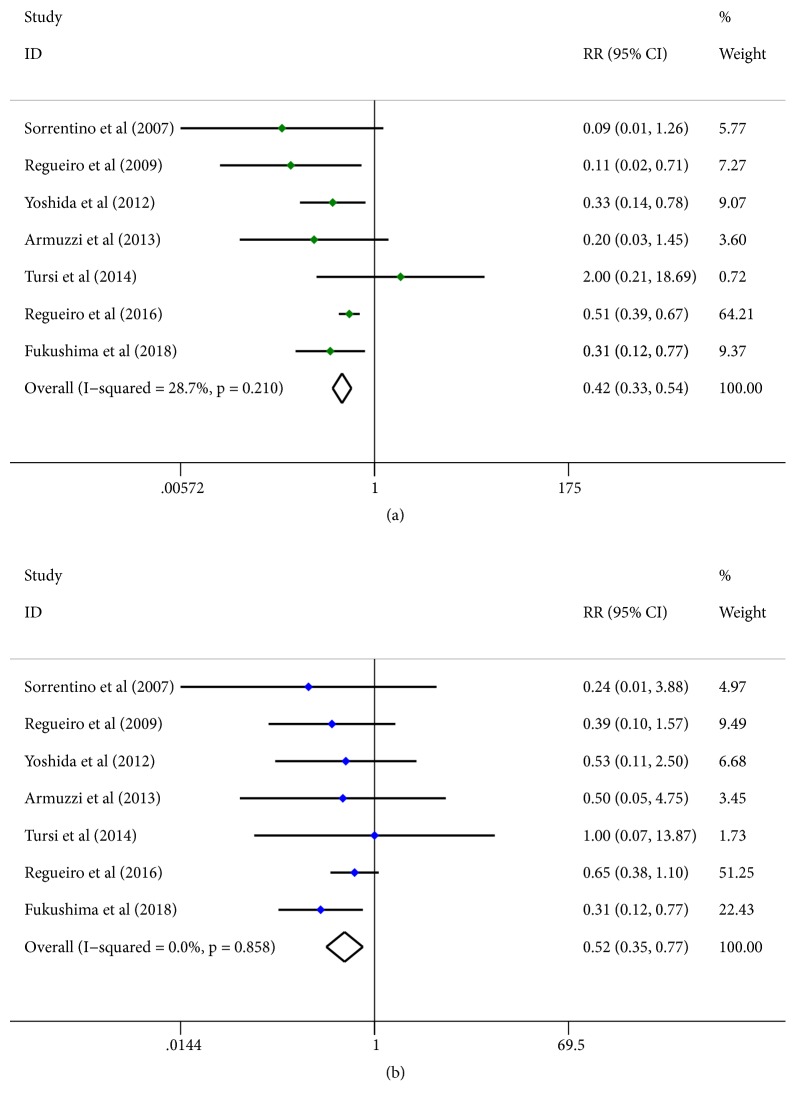
Forest plot of the efficacy of Infliximab in the 7 prospective trials. (a) Rates of endoscopic recurrence. (b) Rates of clinical recurrence. Green dots represent the RR of rates of endoscopic recurrence and blue dots represent the RR of rates of clinical recurrence. Horizontal lines represent 95%Cls of the RR estimates. Diamonds stand for summary effect estimate of the meta-analysis.

Endoscopy is gold standard for monitoring postoperative recurrence and endoscopic recurrence is defined as Rutgeerts score≥2. Higher rates of endoscopic recurrence indicate worse curative effect. In the pooled analysis, Infliximab therapy significantly decreased the rate of endoscopic recurrence of CD compared with a placebo (RR = 0.421; 95% CI 0.328 to 0.539;* p*<0.001; Figure 5(a)). Except for the Tursi et al. (2014) study, all the trials showed a substantial reduction in rates of endoscopic recurrence.

#### 3.4.2. Rates of Clinical Recurrence

As endoscopy is an invasive examination so that it is difficult to be used as a routine means of close follow-up after surgery, clinical examination strategy helps to improve compliance of patients after operation and clinical recurrence is defined as Hanauer>2, CDAI>150, or HBI>8. A random-effect model was used to perform the meta-analysis of the six included prospective trials. There was a significant reduction of the rates of clinical recurrence in the Infliximab-treated group compared with the placebo group (RR =0.519; 95% CI 0.349 to 0.774;* p*=0.001; Figure 5(b)).

### 3.5. Safety of Infliximab Treatment

**Figure 6:**
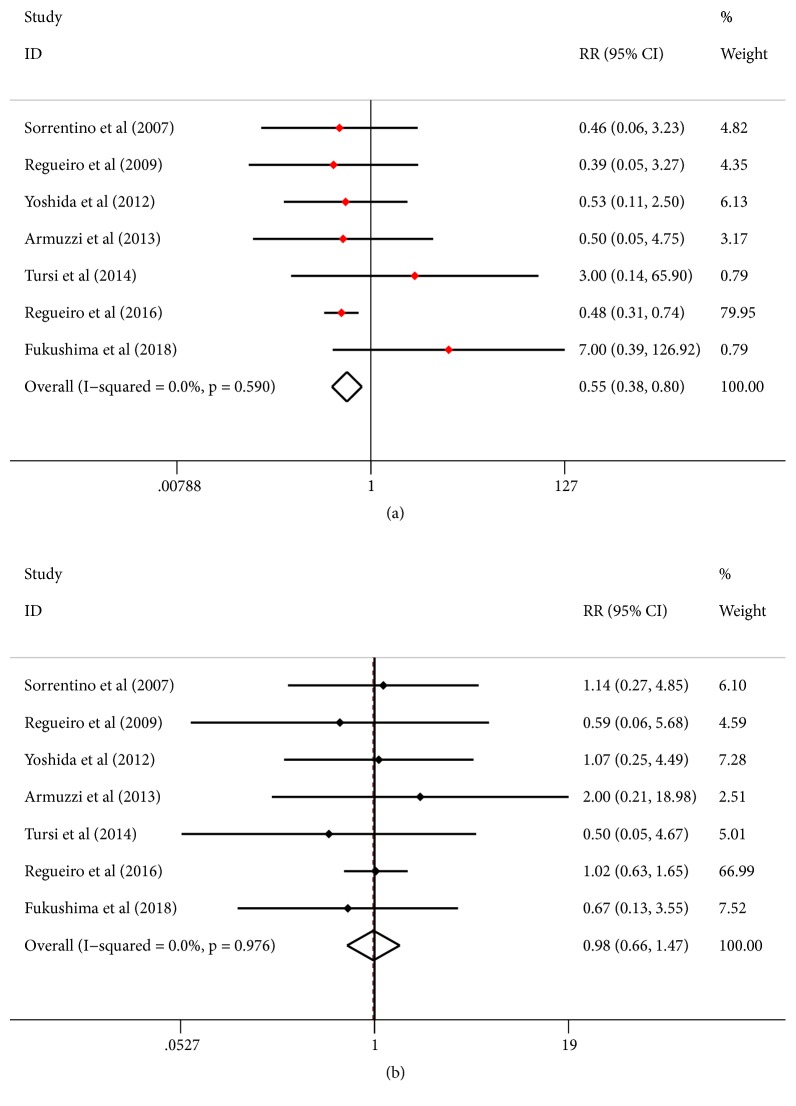
Forest plot of the safety of Infliximab treatment. (a) Rates of adverse events. (b) Rates of study discontinuation. Red dots represent the RR of rates of adverse events and black dots represent the RR of rates of study discontinuation. Horizontal lines represent 95%Cls of the RR estimates. Diamonds stand for summary effect estimate of the meta-analysis.

Overall, Infliximab was well tolerated and has a favorable safety profile that is substantially better than most systemic agents. The most commonly reported adverse events (AE) in patients treated with Infliximab were lupus-like reaction, infusion reaction, bronchitis, nasopharyngitis, pyelonephritis, severe abdominal pain, and abdominal wall abscess. We found that AE occurred at a similar rate among different trials without statistical significance (RR=0.551; 95%CI 0.379-0.800;* p*=0.002, Figure 6(a)). Finally, similar risk of study discontinuation between Infliximab and placebo groups was observed (RR=0.983; 95% CI 0.660-1.466;* p*=0.934, Figure 6(b)).

### 3.6. Test of Heterogeneity

**Figure 7:**
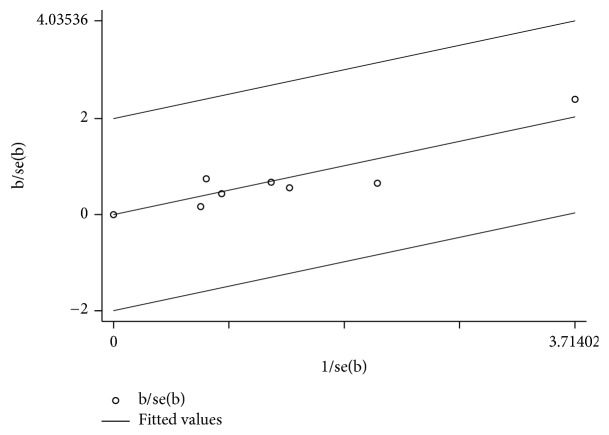
Galbraith radial plot. The figure shows the contribution of results from the 7 studies to the heterogeneity. No study was shown to be the source of the heterogeneity.

We attempted to identify potential sources of heterogeneity in the overall effect based on using forest plots. We discovered that the rates of endoscopic recurrence (I^2^=28.7%, p=0.210) slightly affected the heterogeneity of the outcome result, whereas the rates of clinical recurrence (I^2^=0.0%, p=0.858), the adverse events occurrence (I^2^=0.0%, p=0.590), and the rates of study discontinuation (I^2^=0.0%, p=0.976, Figure 7) were not statistically heterogeneous. To explore the source of heterogeneity, a Galbraith radial plot was performed. The results showed that no study was the major source of heterogeneity.

## 4. Discussion

The results of this meta-analysis demonstrated the efficacy and safety of Infliximab in the prevention of recurrence in patients with CD across 7 prospective trials. Infliximab was shown to be consistently more effective than a placebo. To our knowledge, this is the most recent systematic analysis and comparison of all Infliximab study data for the prevention of CD recurrence.

The trials included had a high level of consistency in the patients enrolled, randomization and masking, and efficacy outcomes. The endpoints these clinical trials applied included both objective and subjective indexes, providing comprehensive assessment of the treatment responses. Endoscopic recurrence is an objective assessment of CD recurrence, whereas clinical recurrence combines both objective and subjective evaluations of the disease recurrence. In most studies clinical recurrence after the resective surgery is characterized by CDAI >220 and in the* Regueiro et al. 2009, Yoshida et al. 2012, *and* Fukushima et al. 2018* studies the criteria are defined as CDAI>150. The 7 trials included in this review were consistent in applying similar objective and subjective recurrence rates, and the results from 7 trials were also consistent, in that Infliximab was effective in the prevention of CD recurrence as evidenced not only by decreased rates of endoscopic recurrence, but also by decreased rates of clinical recurrence.

In our meta-analysis, the sample size was not large enough for valuable statistical results; however, there was a trend towards the efficacy of Infliximab treatment. Further trials should be performed to optimize the dose for the best cost/effect of Infliximab. In addition, a combination of Infliximab with other drugs may improve its effect and thus decrease the dose of Infliximab while achieving similar efficacy. Prospective trials with Infliximab did not identify any serious adverse events. Further clinical trials could provide more information on this issue. Overall, Infliximab is a promising therapeutic agent for improving the management of CD patients. Most importantly, the Infliximab treatment was demonstrated to be very safe.

A limitation of this meta-analysis is the limited number of trials included. However, all the trials were prospective studies and are therefore of high quality. Furthermore, although almost all the publications included in this study were from top journals with high impact factors, risks of bias, e.g., due to funding from the pharmaceutical industry, may exist. Due to ethical limitations, it is difficult to perform clinical trials on healthy people, which play important role in control group, and it would be fine to compare low-risk population (the first resection/nonpenetrating disease) and high-risk CD populations on the efficacy of Infliximab (very relevant for clinical practice); future studies should address the issue in these patient's groups.

Systemic immunosuppressant drugs are still recommended by the current guidelines for the prevention of CD recurrence when topical treatment or phototherapy is ineffective; however, the frequency of serious adverse events hinders the use of systemic anti-inflammation treatment. Our findings clearly indicate that Infliximab is promising as an anti-CD recurrence medication and meets the requirements of treating the disease specifically and efficiently. Further investigations should aim to prove the long-term stability, efficacy, and safety of Infliximab in the treatment of CD.

## Figures and Tables

**Table 1:** The characteristics of the 7 included trials*∗*.

Study	Drug Regimen	Follow-up	Type of Ileocolonic Resection	Timing of Initiation of Treatment	Recurrence Definition	Patients(n)	Endoscopic Recurrence (%)	Clinical
		(month)						Recurrence (%)
Sorrentino et al.	IFX 5mg/kg	24	Macroscopically diseased bowel resected	2 weeks postresection	Clinical:Hanauer>2,Endoscopic: Rutgeerts scor≥2	7	0(0)	0(0)
	MES2.4g/d					16	12(75)	4(25)
Regueiro et al.	IFX 5mg/kg	12	Macroscopically diseased bowel resected	within 4 weeks of resection	Clinical:CDAI>150,Endoscopic: Rutgeerts scor≥2	11	1(9)	2(18)
	Placebo					13	11(85)	6(46)
Yoshida et al.	IFX 5mg/kg	12	Macroscopically diseased bowel resected	4 weeks postresection	Clinical:CDAI>150,Endoscopic: Rutgeerts scor≥2	15	4(26)	2(13)
	No treatment					16	13(81)	4(25)
Armuzzi et al.	IFX 5mg/kg	12	Curative ileocolonic resection	2-4 weeks postresection	Clinical:HBI>8,Endoscopic: Rutgeerts scor≥2	11	1(9)	1(9)
	AZA 2.5mg/kg/d					11	5(45)	2(18)
Tursi et al.	IFX 5mg/kg	12	Curative ileocolonic resection	within 4-6 weeks of resection	Clinical:HBI>8,Endoscopic: Rutgeerts scor≥2	10	2(20)	1(10)
	ADA 40mg EOW					10	1(10)	1(10)
Regueiro et al.	IFX 5mg/kg	12	Macroscopically diseased bowel resected	within 6 weeks of resection	Clinical:CDAI>200,Endoscopic: Rutgeerts scor≥2	147	45(31)	19(13)
	Placebo					150	90(60)	30(20)
Fukushima et al.	IFX 5mg/kg	24	Curative ileocolonic resection	within 8 weeks of resection	Clinical:CDAI>150,Endoscopic: Rutgeerts scor≥2	19	4(21)	4(22)
	Placebo					19	13(68)	13(69)

*∗* IFX, infliximab; MES, mesalamine; ADA, adalimumab; AZA, azathioprine; CDAI, Crohn's disease activity index; EOW, every other week; HBI, Harvey-Bradshaw index.
